# T cells in acute and chronic myocarditis: from diagnosis to treatment

**DOI:** 10.1093/eurheartj/ehaf1080

**Published:** 2026-01-29

**Authors:** Evelyn J Song, Anna Joachimbauer, Sofia Tasca, Richard Baylis, Dörthe Schmidt, Burkhard Ludewig, Javid J Moslehi

**Affiliations:** Section of Cardio-Oncology & Immunology, Cardiovascular Research Institute (CVRI), University of California San Francisco, 555 Mission Bay Blvd S, San Francisco, CA 94158, USA; Division of Cardiology, Department of Medicine, University of California San Francisco, San Francisco, CA, USA; Institute of Immunobiology, Cantonal Hospital St. Gallen, Rorschacher Strasse 95, CH-9007, St. Gallen, Switzerland; Department of Cardiology, University Heart Center, University Hospital Zurich, University of Zurich, Rämistrasse 100, CH-8091, Zurich, Switzerland; Section of Cardio-Oncology & Immunology, Cardiovascular Research Institute (CVRI), University of California San Francisco, 555 Mission Bay Blvd S, San Francisco, CA 94158, USA; Section of Cardio-Oncology & Immunology, Cardiovascular Research Institute (CVRI), University of California San Francisco, 555 Mission Bay Blvd S, San Francisco, CA 94158, USA; Department of Cardiology, University Heart Center, University Hospital Zurich, University of Zurich, Rämistrasse 100, CH-8091, Zurich, Switzerland; Institute of Immunobiology, Cantonal Hospital St. Gallen, Rorschacher Strasse 95, CH-9007, St. Gallen, Switzerland; Department of Cardiology, University Heart Center, University Hospital Zurich, University of Zurich, Rämistrasse 100, CH-8091, Zurich, Switzerland; Center for Translational and Experimental Cardiology (CTEC), Department of Cardiology, University Hospital Zurich, University of Zurich, Wagistrasse 12, CH-8952 Schlieren, Zurich, Switzerland; Section of Cardio-Oncology & Immunology, Cardiovascular Research Institute (CVRI), University of California San Francisco, 555 Mission Bay Blvd S, San Francisco, CA 94158, USA

**Keywords:** Myocarditis, T lymphocytes, Adaptive immune response, Immunosuppression, Cardio-oncology

## Abstract

Myocarditis refers to infiltration of immune cells into the heart causing inflammation and cardiomyocyte damage. Clinically, myocarditis can be acute or chronic. While the clinical and pathological syndrome of myocarditis has been recognized for more than a century, newer aetiologies for myocarditis have emerged in the past decade. These include myocarditis associated with immune checkpoint inhibitors, or myocarditis linked to chronic inflammatory and genetic diseases. With the emergence of immune checkpoint inhibitor-associated myocarditis, the breakdown of T cell tolerance has been recognized as a key mechanism in disease development. The main focus of this review is to integrate existing models of myocarditis into an overarching immunological concept. Through the lens of loss of immune tolerance, this review focuses on diagnosis and treatment of myocarditis. Starting from acute myocarditis as paradigmatic inflammatory condition of the heart, this review outlines future research frontiers for myocardial inflammatory disease including new approaches to diagnosis and treatment.

## Graphical Abstract

**Graphical Abstract:**
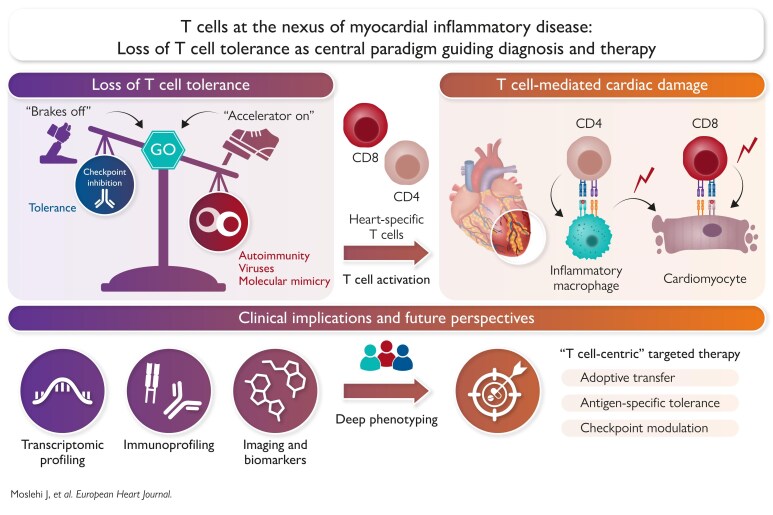
T cell tolerance and activation as key drivers of myocarditis.

## Introduction

Myocardial inflammatory disease is characterized by the infiltration of immune cells into the heart, leading to a wide range of symptoms and disease manifestations^1,2^ that are included in the inflammatory myopericardial syndrome.^3^ More specifically, myocarditis, the clinical consequence of myocardial inflammation, can range from mild to fulminant in the acute phase and stable, recurrent, or progressive in the chronic phase.^3,4^ Acute myocarditis and its long-term sequelae, such as chronic myocarditis or inflammatory cardiomyopathy, are prototypical cardiac conditions that highlight the detrimental effects of excessive myocardial immune cell activity.^5^

**Figure 1:**
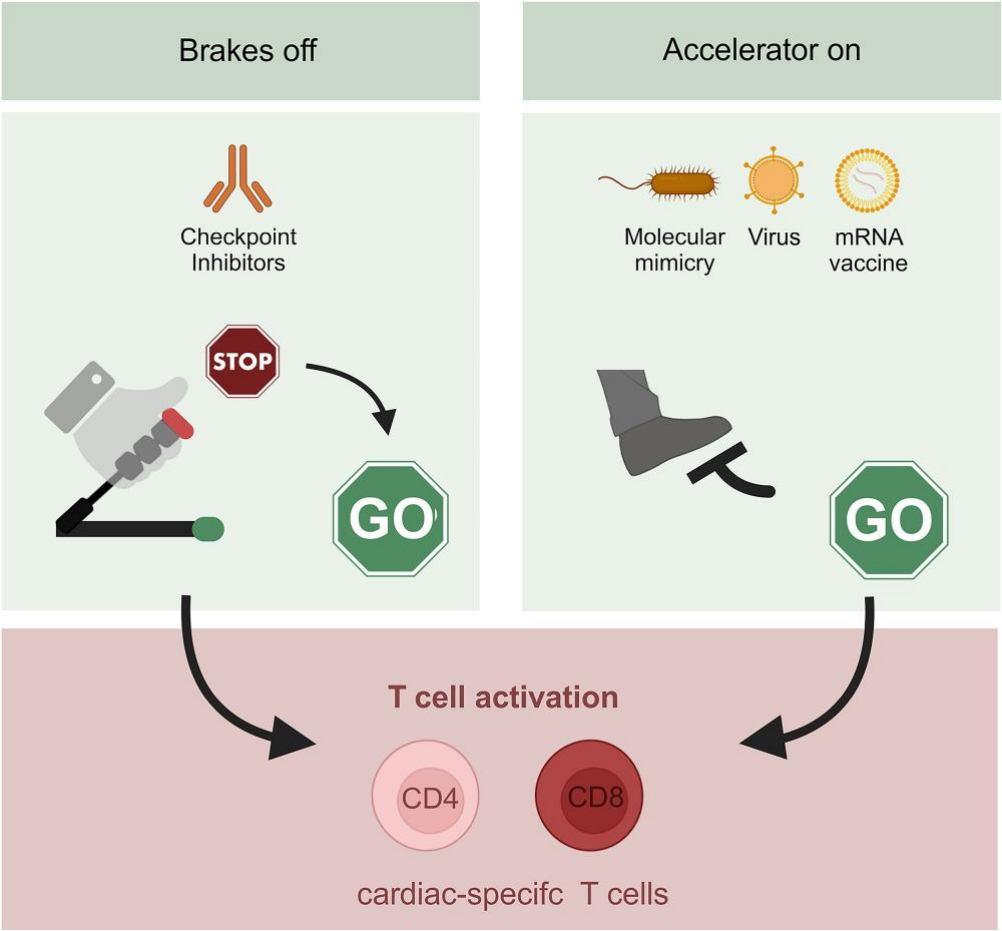
T cell activity at the nexus of myocardial inflammatory disease. Immune checkpoint inhibition attenuates processes that limit the activity of heart-specific T cells (‘brakes off’), whereas stimulatory signals from antigenic mimicry or global immune activation during infection or vaccination enhance and accelerate tissue-destructive T cell activity (‘accelerator on’)

The emergence of new forms of myocarditis over the past decade highlights the central and critical role that T lymphocytes play in the development and propagation of myocarditis and other myocardial diseases.^6,7^ Perhaps most informative has been the occurrence of myocarditis associated with novel cancer therapies, called immune checkpoint inhibitors (ICIs).^8,9^ The pattern of disease onset in ICI myocarditis suggests that (i) complex, multi-layered tolerance mechanisms prevent autoimmune-mediated damage of the heart and (ii) immune cell activation and the resulting myocardial damage can occur rapidly and extensively. Since ICIs are designed to attenuate immune mechanisms that restrain T cell activity, experimental models of ICI myocarditis as well as data from affected patients suggest that heart-specific T cells are the main drivers of tissue-destructive processes in the myocardium (*Figure 1*). More broadly, excessive activation of heart-specific T cell driven by bacterial structures mimicking cardiac antigens or the global immune activation during viral infection or vaccination can boost and accelerate damage to the cardiac tissue (*Figure 1*).

This review integrates virus- and pathogen-centric models into an overarching immunological framework centred on T cell tolerance and activation as key drivers of myocarditis. Through the lens of T cell function and regulation, we connect current clinical approaches with mechanistic insights and identify critical knowledge gaps to guide future diagnostic and therapeutic advances.

## Pathogenesis of myocarditis: a breakdown in immune tolerance

The immune system is designed to distinguish between non-infectious self and infectious non-self.^10^ While the innate arm of the immune system uses germline-encoded receptors to detect conserved immunogenic patterns and structures of pathogens (e.g. bacterial lipopolysaccharides), the adaptive immune system uses genetically modifiable receptors [i.e. antibodies produced by B cells and T cell receptors (TCRs) expressed by T cells] to recognize variable antigenic structures.^11,12^ The high diversity of the TCR repertoire facilitates recognition of a large fraction of pathogen-derived antigens displayed by the major histocompatibility complex (MHC).^13^ However, the broad spectrum of TCR reactivity carries the risk that self-antigens and harmless commensal-derived antigens are recognized by T cells and cause autoimmune inflammatory diseases. In addition, emerging data suggest that abnormal T cell regulation plays a critical role in development of myocarditis, i.e. the loss of immune tolerance to cardiac antigens (*Figure 1*).

### Development and differentiation of heart-specific T cells

**Figure 2:**
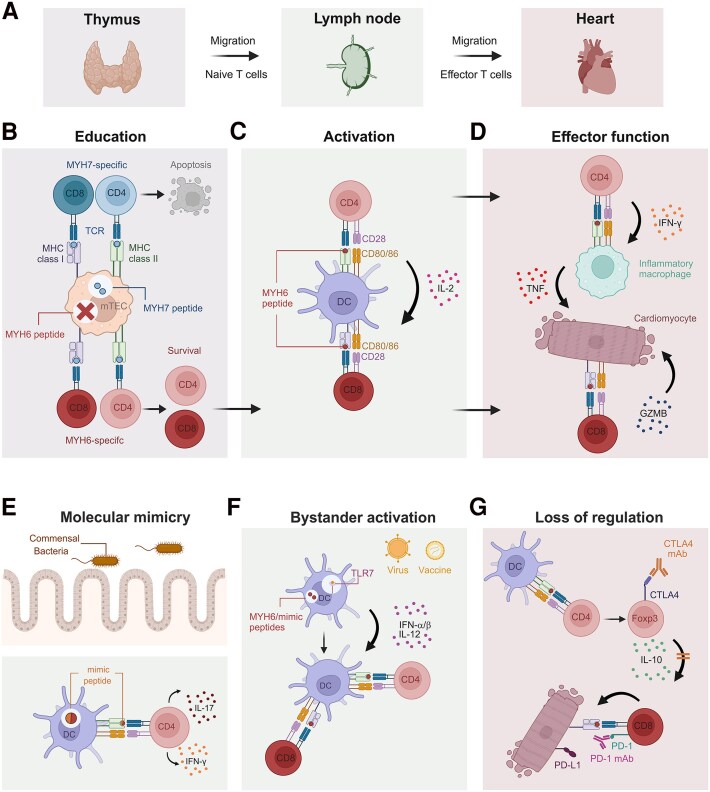
Activation and control of heart-specific T cells. (*A*) T cells undergo education in the thymus and circulate as naive T cells through lymph nodes. Upon activation, they clonally expand and migrate as effector T cells to peripheral tissues. (*B*) In the thymus, T cells are educated to distinguish self from non-self. Recognition of self-peptides presented by medullary thymic epithelial cells (mTECs), such as the cardiac protein myosin heavy chain 7 (MYH7), triggers apoptosis. Due to the lack of thymic expression, T cells specific for the cardiac protein myosin heavy chain 6 (MYH6) escape the thymus. (*C*) In heart-draining lymph nodes, MYH6-specific T cells are activated by recognizing the MYH6–MHC complex on dendritic cells and receiving costimulatory signals (CD28-CD80/CD86 interaction). Concomitant antigen presentation via MHC class I and II activated CD8^+^ T cells, with CD4^+^ T cells providing help via interleukin-2 (IL-2). Following activation and expansion, effector T cells migrate to the heart. (*D*) Re-encountering cardiac antigens in the heart, effector T cells induce cardiomyocyte damage. CD4^+^ T cells release interferon-gamma (IFN-γ) to activate inflammatory macrophages, which in turn induce tumour necrosis factor (TNF)-mediated tissue damage. CD8^+^ T cells directly damage cardiomyocytes through the release of cytotoxic molecules, such as granzyme B (GZMB). (*E*) Molecular mimicry occurs when bacterial-derived mimic peptides, structurally similar to the cardiac protein MYH6, activate cross-reactive MYH6-specific CD4^+^ T cells. These T cells produce IFN-γ, triggering activation of inflammatory macrophages and leading to cardiac damage. (*F*) Infection or immunization activates the innate immune system by stimulating pattern recognition receptors such as TLR7, which recognizes viral RNA. Upon TLR7 stimulation, dendritic cells release type I interferons (IFNα/β) and IL-12, enhancing their antigen presentation capacity. Inflammation-induced activation of cardiac-specific T cells through dendritic cells presenting MYH6 or mimic peptides is referred to as bystander activation. (*G*) Regulatory mechanisms are important for preventing excessive T cell activation and tissue damage. CD4^+^ T cell activation leads to increased expression of the co-inhibitory molecule CTLA4 and upregulation of Foxp3 in activated CD4^+^ T cells, promoting regulatory T cell functions. Elevated PD-L1 expression in inflamed tissue controls CD8^+^ T cell cytotoxicity. Immune checkpoint inhibitors (antibodies against PD-1/PD-L1 and/or CTLA4) remove the regulatory ‘brake’ on MYH6-specific T cells and trigger autoimmune responses against the heart. TCR, T cell receptor; MHC, major histocompatibility complex; IL-2, interleukin 2; IFN-γ, interferon γ; TNF, tumour necrosis factor; DC, dendritic cell; GZMB, granzyme B; TLR7, toll-like receptor 7; IFN-α/β, interferon α/β; IL-12, interleukin 12; IL-10, interleukin 10; CTLA4, cytotoxic T lymphocyte-associated protein 4; mAb, monoclonal antibody; PD-1, programmed cell death protein 1; PD-L1, programmed-cell death ligand 1

T cell progenitors are generated in the bone marrow and educated in the thymus before migrating as naive T cells into lymph nodes, where they are clonally expanded and programmed to become effector T cells that migrate into the tissue (*Figure 2A*). Self-reactive T cell clones recognizing tissue antigens are deleted in the thymus.^14–16^ Several cardiac antigens such as myosin heavy chain 7 (MYH7) or cardiac troponin T (TNNT2) are expressed by medullary thymic epithelial cells^17^ leading to the elimination of reactive T cell clones by apoptosis (*Figure 2B*). In contrast, cardiac proteins such as MYH6 are not expressed in the thymus^17–19^ facilitating the exit of MYH6-specific T cells from the thymus (*Figure 2B*)^17,20^ and their migration to heart-draining lymph nodes.^18,21^ The activation of naive T cells and their expansion in lymph nodes requires recognition of peptide–MHC complexes on dendritic cells in combination with costimulatory signals such as CD28 binding to CD80/CD86 molecules (*Figure 2C*). Accordingly, the lack of CD28 ameliorates experimental autoimmune myocarditis.^22^ Concomitant presentation of MHC class I and II-bound peptides by the same dendritic cell bolsters cross-presentation of antigen and activation of CD8^+^ T cells^23^ via interleukin-2 (IL-2) provided by CD4^+^ T cells^24^ (*Figure 2C*). Following expansion and the acquisition of effector functions in the lymph node environment, heart-specific T cells migrate towards their target tissue, where they cause cardiomyocyte damage (*Figure 2D*). CD4^+^ T effector cells in the myocardium produce interferon-gamma (IFN-γ) following recognition of cardiac antigen presented by cardiac dendritic cells^25,26^ and thereby foster cardiomyocyte-damaging activity of inflammatory macrophages^21^ (*Figure 2D*). CD8^+^ T cells, on the contrary, can directly damage cardiomyocytes by releasing cytotoxic molecules such as granzyme B^27^ (*Figure 2D*). Taken together, the development and differentiation of heart-specific T cells are tightly controlled through multi-layered cellular and molecular processes. T cell-mediated damage to the myocardium results from aberrant stimulation of heart-specific T cells or from the loss of distinct regulatory circuits.

### Molecular mimicry

The vast number of microbial antigenic peptides exceeds the number of recombined TCRs, resulting in the recognition of multiple, structurally related peptides by the same TCR.^13^ This basic principle of TCR cross-reactivity is also known as molecular mimicry.^28^ Myocardial inflammatory disease has been suggested to result from cross-reactivity with pathogen-derived antigen.^29,30^ However, the question to what extent TCR cross-reactivity against pathogenic viruses contributes to autoimmune disease has not been resolved yet.^31,32^ Notably, the commensal microbiome harbours peptides that mimic self-antigens, as demonstrated for β-galactosidase peptides derived from *Bacteroides* species that are recognized by MYH6-specific T cells.^21^ Activation of cross-reactive MYH6-specific CD4^+^ T cells by such mimic peptides can foster activation of inflammatory macrophages in the heart and exacerbation of cardiac damage (*Figure 2E*). Further in-depth analyses are required to fully characterize the cross-reactivity of heart-specific TCRs and their impact on acute myocarditis and other inflammatory conditions of the myocardium.

### Infection-associated bystander activation

Patients presenting with acute myocarditis frequently report prior viral infection and infection-associated symptoms.^1,33^ However, endomyocardial biopsies in paediatric and adult myocarditis are frequently virus-negative.^34^ Moreover, it remains unclear to what extent ubiquitous viruses that are present in the myocardium of healthy individuals, such as Parvovirus B19,^35^ contribute to inflammatory myocardial disease. Likewise, the role of lymphotropic viruses such as human herpes virus 4 and 6 in myocarditis has yet to be elucidated.^36^ Current consensus on the involvement of viral infection in myocarditis pathogenesis implies that systemic spread of the virus rather than replication of the virus in the heart tissue itself is the key trigger for the disease.^5,37^ Accordingly, systemic triggering of pathogen recognition receptors such as the viral RNA receptor TLR7 activates the innate immune system and promotes myocardial inflammation^38^ (*Figure 2F*). Infection-triggered TLR7 stimulation of dendritic cells fosters production of type I interferons (IFN-α/β) and IL-12, which increase the antigen presentation capacity of dendritic cells including cross-presentation to CD8^+^ T cells^39^ (*Figure 2F*). Viral infection with systemic release and distribution of inflammatory mediators is commonly referred to as ‘cytokine storm’, as observed in SARS-CoV-2 and influenza infection.^40,41^ The efficacy of mRNA vaccines to induce CD8^+^ T cell responses in the spleen depends on type I interferon and activation of macrophages.^42^ It is thus conceivable that, for example, SARS-CoV-2 vaccination-associated myocarditis is driven by the same processes that cause virus infection-enhanced activation of heart-specific T cells (*Figure 2F*). The low incidence and mild course of vaccine-associated myocarditis compared with SARS-CoV-2 infection^43^ suggests that the relatively low level of systemic bystander activation during vaccination induces only a weak activation of heart-specific T cells compared with the strong cytokine storm characteristic of severe viral infection. Thus, the protective effect of vaccination against SARS-CoV-2 greatly outweighs the relatively small risk of developing usually a mild myocarditis as a side effect.^43^

### Loss of immune regulation

T cell activation involves the induction of intrinsic regulatory mechanisms that prevents overshooting proliferation and excessive tissue damage.^44^ Such dynamic tuning of CD4^+^ T cell activity during their activation in lymph nodes and during the effector phase in the tissue involves increased expression of co-inhibitory molecules such as CTLA4 and control of T cell proliferation via the transcription factor Foxp3^45^ (*Figure 2G*). Multilevel control of CD8^+^ T cell activity is achieved to some extent through the provision of immune-attenuating cytokines such as IL-10 and TGF-β (transforming growth factor beta) by regulatory T cells^46,47^ and enhanced expression of the PD-1 by effector T cells in the target tissue, which promotes their exhaustion and thereby protects the tissue from immunopathological damage.^48^ Inflamed tissues react with increased expression of PD-1 ligands to ameliorate potentially harmful CD8^+^ T cell activity.^49^ Consequently, mice lacking the expression of the critical co-inhibitory molecules CTLA4 and PD-1 develop multi-organ inflammation including myocarditis.^50,51^ Cancer patients treated with antibodies directed against the immune checkpoints PD-1/PD-L1 and/or CTLA4 develop a broad range of autoimmune side effects including myocarditis.^52^ The ICI myocarditis is a rare but potentially severe complication with a mortality rate of up to 50%^8,53,54^ with self-reactive T cells being directed against MYH6.^20,37^ The presence of MYH6-specific T cells in the circulation of healthy individuals highlights that the immune system has to employ elaborate regulatory mechanisms to restrain the activity of heart-specific T cells^6^ (*Figure 2G*). Autoreactive CD4^+^ and CD8^+^ T cells recognizing MYH6 and hitherto unknown self-antigens^55^ are probably the cause for the rapid onset of myocarditis after ICI treatment with antibodies against CTLA4, PD-1, and/or PD-L1.^52^ However, the risk factors and immunological mechanisms that predispose certain cancer patients to ICI myocarditis while sparing others are still largely elusive. Therefore, further elaboration of the basic mechanisms underlying T cell-mediated myocardial inflammation is warranted to allow for patient stratification and optimization of anti-inflammatory treatment of acute and chronic myocarditis.

## Clinical presentation and disease course

**Figure 3:**
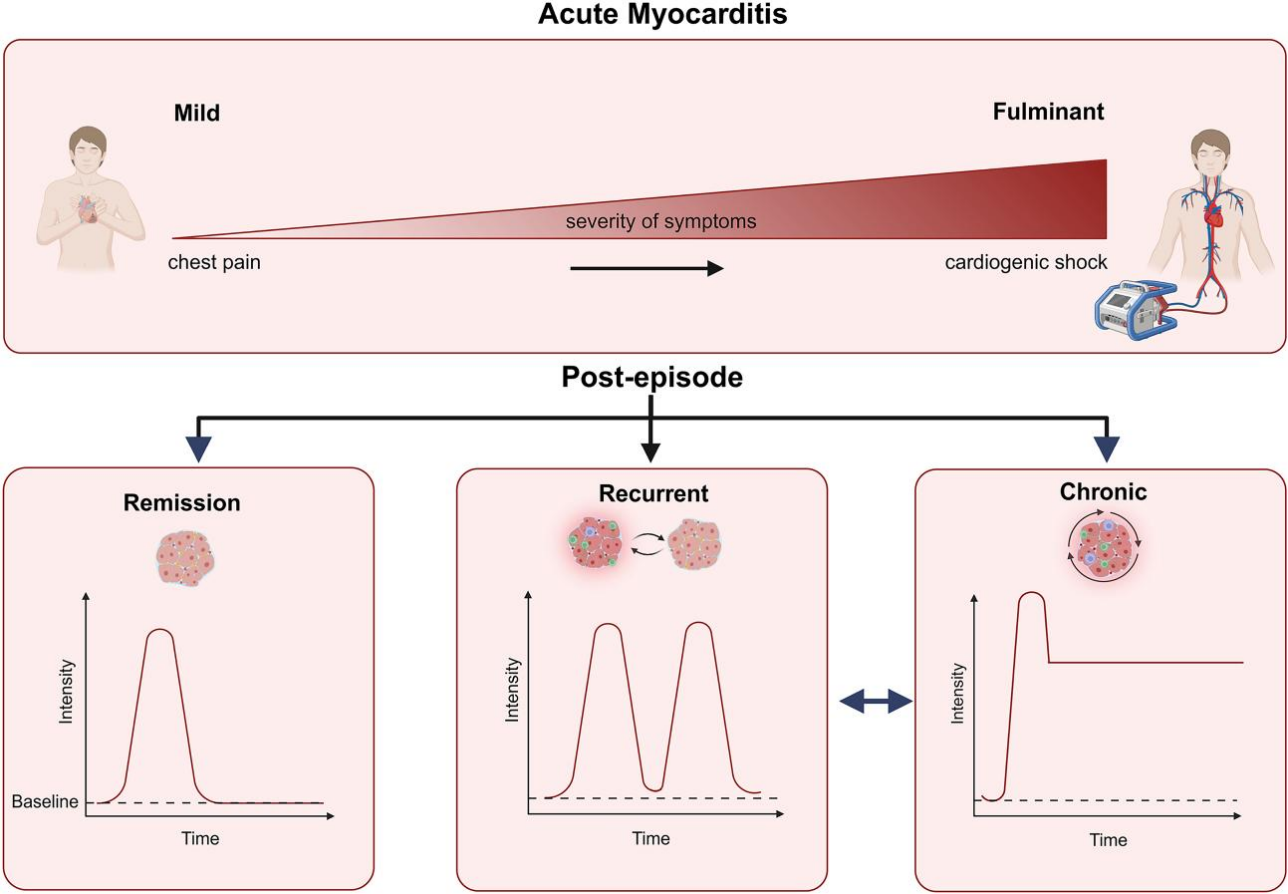
Clinical characterization of myocarditis. Patients with acute myocarditis can present with a range of symptoms, with 3%–9% progressing to acute fulminant myocarditis, characterized by its rapid onset and progression of symptoms. Following the resolution of acute symptoms, patients enter the post-episodic phase which can lead to one of three outcomes: resolution, chronic myocarditis, or recurrent myocarditis. Recurrent myocarditis requires more than one acute episode requiring hospitalization. Chronic myocarditis is persistent cardiac inflammation lasting for more than 30 days from the initial onset of symptoms. Resolution is defined as complete recovery, including symptom relief, biomarker normalization, and resolution of structural abnormalities

Recognizing T cell-driven myocardial inflammation from diverse triggers is key to mechanism-based clinical phenotyping. Acute myocarditis, either following an episode of infection or initiation of ICI treatment, manifests within a few weeks.^53,56^ Mild forms of the disease resolve completely within 30 days. However, a subset of patients (around 3%–9%) presents with acute fulminant myocarditis (FM), characterized by a rapid onset and progression to severe complications such as cardiogenic shock or life-threatening arrhythmias^57,58^ (*Figure 3*). The mortality rates in FM are high, especially in ICI myocarditis, reaching 50%.^53,54,59^ Following initial presentation with acute myocarditis, patients’ outcomes vary. Some recover fully without recurrence or residual cardiac dysfunction or experience recurrent myocarditis episodes, requiring hospitalizations (*Figure 3*). For some, myocarditis could progress to chronic myocarditis, defined by persistent myocardial inflammation lasting more than 3 months.^3,4^ While inflammatory cardiomyopathy is characterized by chronic inflammation with persistent cardiac dysfunction and ventricular remodelling (dilated or non-dilated morphology), chronic myocarditis remains ill-defined.^3,4^ It is still uncertain whether chronic myocarditis represents an intermediate disease stage between acute myocarditis and inflammatory cardiomyopathy or constitutes a distinct disease entity marked by ongoing cardiac inflammation in the absence of cardiac dysfunction (*Figure 3*). Responsiveness to cardiac myosin antigens in patients with chronic disease suggests that persistent cardiac inflammation can be driven by autoimmune T cell-mediated processes.^60,61^

Estimating the true prevalence of myocarditis is challenging, as many cases go undiagnosed. Globally, it is thought to range from about 10.2 to 105.6 cases per 100 000 people, translating to roughly 1.8 million new cases each year.^62^ While the clinical manifestations of myocarditis vary substantially, common patterns are clearly distinguishable. Chest pain is the most prevalent symptom, reported in approximately 82%–95% of cases.^4,57,63–65^ Other common symptoms include fever, occurring in 58%–65% of patients, dyspnoea (19%–49%), and syncope (5%–7%).^1,57^

There are sex differences in the median age of diagnosis. In men, the median age ranges from 30 to 45 years, while in women, it spans from 42 to 45 years.^63,66^ Acute myocarditis exhibits a higher prevalence in males,^63,66^ while sex differences in ICI myocarditis are less pronounced in younger patients but may pose a relevant risk factor in elderly.^67,68^ Notably, patients presenting with a left ventricular ejection fraction (LVEF) < 50% face a four-fold increased risk of adverse cardiovascular events, including acute decompensated heart failure, ventricular arrhythmias, and in-hospital mortality.^63^ Risk enhancers in ICI myocarditis include the presence of an active thymoma, concomitant myositis, and a reduced LVEF < 50%, which are associated with increased risk of 30-day mortality.^69^ In COVID-19 vaccine-related myocarditis, males aged 12–30 years have the highest risk and symptoms typically begin within 1–14 days post vaccination after the second dose of the primary series of vaccination with an mRNA vaccine.^70^

This diverse clinical presentation underscores the importance of a high index of suspicion for myocarditis in patients presenting with these symptoms, without an alternative explanation, particularly when accompanied by recent exposure to potential triggers such as infection, immunotherapy, or when systemic signs suggest systemic immune activation. Therefore, early recognition, appropriate diagnostic evaluation, and disease mechanism-based classification are crucial for optimal patient outcomes.

## Diagnostic workup

**Figure 4:**
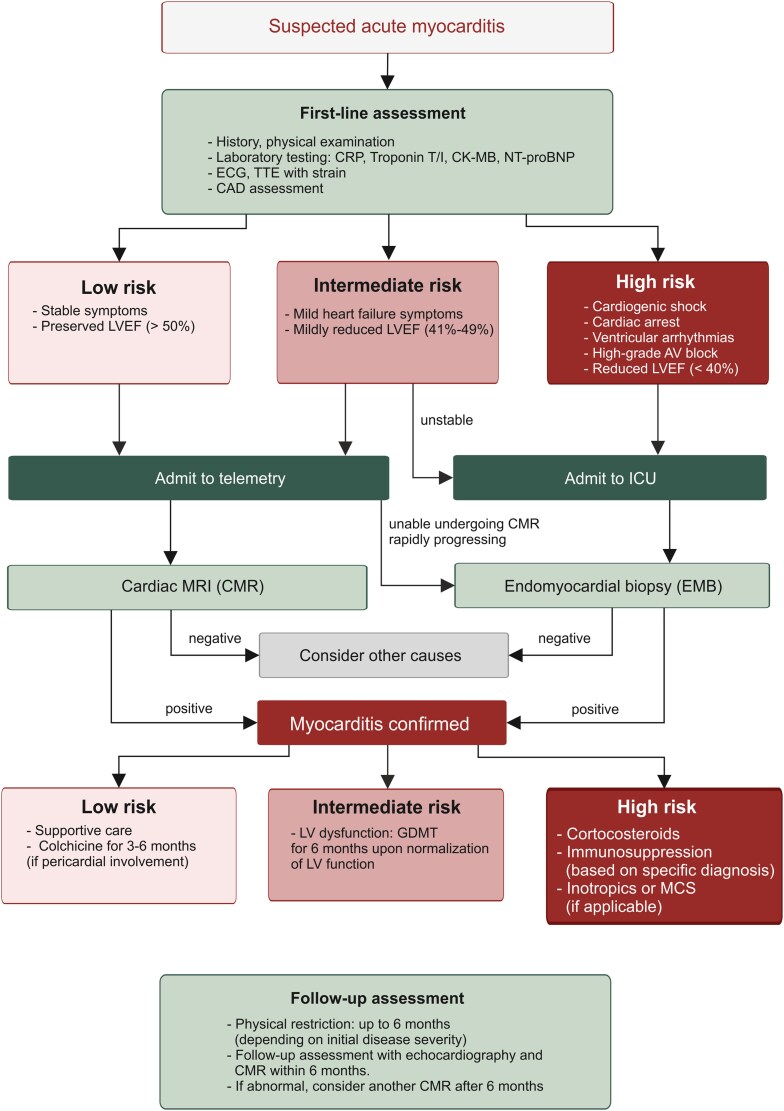
Overview algorithm for the diagnosis and management of acute myocarditis based on 2025 ESC guidelines. CRP, C-reactive protein; CK-MB, creatinine kinase MB; NT-BNP, N-terminal-B-type natriuretic peptide; ECG, electrocardiogram; TTE, transthoracic echocardiogram; CAD, coronary artery disease; LVEF, left ventricular ejection fraction; ICU, intensive care unit; CMR, cardiac magnetic resonance; GDMT, guideline-directed medical therapy; MCS, mechanical circulatory support

Myocarditis should be considered in patients presenting with compatible symptoms and history, alongside with distinct clinical findings in the first-line assessment such as laboratory evidence of myocardial injury and inflammation or abnormal ECG.^3^ Because of overlapping symptoms, obstructive coronary artery disease must be excluded promptly^71^ (*Figure 4*), which could be achieved by novel biomarkers.^72^ However, the markers of the current first-line assessment lack specificity, and normal values do not rule out myocarditis.^73^ High-sensitivity troponin I or T levels are elevated in approximately 64%–100% of myocarditis cases, while CRP levels are elevated in 54%–99%.^1^ In ICI myocarditis, elevated troponin T levels correlate with worse outcomes and provide greater sensitivity for patient monitoring.^74^ In addition, neutrophil-lymphocyte ratio (NLR) may serve as a potential biomarker with high NLR indicating a worse prognosis.^75,76^ Although viral serology may indicate a recent viral illness, it lacks sensitivity and specificity and is not recommended for diagnosing myocarditis in recent guidelines.^3,4^

### Echocardiography

Echocardiography is essential for the evaluation of risk profiles in the first-line diagnostic workup, enabling a rapid assessment of cardiac function and the exclusion of alternative causes of symptoms. A significantly reduced LVEF offers prognostic insights and guides initial triage decisions^3,57^ (*Figure 4*). Whenever possible, global longitudinal strain (GLS) should be obtained.^77,78^ Early in the disease course, left ventricular dimensions may appear normal with preserved LVEF but can deteriorate rapidly within the first few days after presentation.^57^

### Cardiac magnetic resonance

Cardiac magnetic resonance (CMR) is currently considered the gold standard for diagnosing myocarditis for stable patients without cardiogenic shock or unstable arrhythmias^3^ (*Figure 4*). The updated 2018 Lake Louise criteria for diagnosing myocarditis rely on detecting myocardial oedema (T2-weighted imaging) and non-ischaemic myocardial injury (T1 mapping, ECV, or LGE), achieving a sensitivity of 87.5% and specificity of 96.2% for acute myocarditis.^79,80^ Additionally, the pattern and location of LGE on CMR provide valuable prognostic information. Specifically, mid-wall and anteroseptal LGE patterns have been associated with a worse prognosis.^73^ Nevertheless, in cases of chronic myocardial inflammation, the sensitivity of 62% is considered as insufficient for diagnostic purposes.^81^ Diagnostic accuracy varies based on the clinical presentation and timing.^82,83^ Despite the value of CMR in the diagnosis of myocarditis, it remains under-used.^84^ Accessibility remains a major limitation—CMR availability is restricted in many regions, particularly in low- and middle-income countries, due to cost, limited scanner capacity, and need for specialized interpretation. Even in experienced centres, interobserver variability in interpreting subtle T1/T2 mapping changes can impact diagnosis.^85^ Ongoing research also aims to identify novel tracers and imaging modalities for the diagnosis of myocarditis.^86,87^

### Endomyocardial biopsy

In high-risk patients presenting with cardiogenic shock, high-grade heart block, and malignant ventricular arrhythmias, endomyocardial biopsy (EMB) remains the recommended diagnostic modality. The detection of excessive accumulation of inflammatory cells in EMB confirms the diagnosis of acute myocarditis^3,71,88,89^ (*Figure 4*), which is based on the presence of inflammatory infiltration, myocyte necrosis in a non-ischaemic pattern, and immune cells adjacent to necrotic myocytes.^90^ Extending the Dallas criteria through immunohistochemical staining for immune cell populations helps to classify the type of myocarditis based on histological findings of inflammatory cells.^91^ For instance, lymphocytic myocarditis is identified by infiltrates of T cells and macrophages,^92,93^ eosinophilic myocarditis by the presence of eosinophils alongside lymphocytes, and giant cell myocarditis by extensive T lymphocyte infiltrates, multinucleated cells, and eosinophils.^94^ Although high viral loads of certain viruses such as enteroviruses, HHV4/6 and PVB19 have been detected in the myocardium of acute myocarditis patients, the interpretation of viral genome detection remains controversial in most cases, as the majority of EMB specimens from acute myocarditis patients is virus-negative.^34^ The detection of viral genomes in healthy cardiac tissue further complicates interpretation, as the mere presence of viral material does not imply a causal relationship.^35^ Recent multicentre data demonstrated that early EMB was associated with reduced rates of death, heart transplantation, and left ventricular assist device implantation at one year compared with delayed EMB underscoring the importance of prompt EMB in patients with FM.^95^ However, it is important to emphasize that EMB has limited sensitivity due to sampling errors resulting from the patchy distribution of myocardial inflammation.^81,96,97^ Sensitivity improves with electrogram-guided or repeated biopsies, but these are rarely performed outside of specialized centres.^96,98^ Procedural risks (tamponade, arrhythmia, tricuspid injury) are low in expert hands but still perceived as barriers, particularly in centres without high procedural volume.^89,91^ Additionally, turnaround time for histology, variable immunohistochemistry protocols, and lack of widespread molecular viral testing further limit diagnostic utility. Given the lack of a single definitive test, accurate diagnosis requires clinical integration of multiple data points, including patient history, laboratory tests, and multimodal imaging.

### Genetic testing

Emerging data indicate that there is a substantial overlap between genetic cardiomyopathies and myocarditis, with arrhythmogenic cardiomyopathy potentially presenting with myocarditis-like episodes (‘hot phases’) in its early stages. Genetic variants are commonly found in patients with myocarditis.^99,100^ The most frequently implicated genes included *TTN* (titin), associated with dilated cardiomyopathy, and *DSP* (desmoplakin), linked to arrhythmogenic cardiomyopathy. The presence of mutations is also associated with worse clinical outcomes.^99,101,102^ The likelihood of genetic cardiomyopathy is even higher in recurrent acute myocarditis.^103^ Therefore, it is important to highlight that the first manifestations of an underlying genetic cardiomyopathy may be myocarditis.^104^ In addition, genetic predisposition in terms of susceptibility to T cell-driven myocardial damage, i.e. the composition of MHC molecules, needs to be considered. For example, the spontaneous development of progressive myocarditis in transgenic mice expressing particular human MHC class II molecules^17,51,105^ supports the notion that MHC class II-dependent antigen presentation to CD4^+^ T cells is a key process that drives myocardial inflammation under various conditions.^60,106^ Moreover, the development of myocarditis in ICI-treated cancer patients with particular MHC antigens^8,20^ underscores that the genetic component underlying antigen presentation to cardiopathogenic T cells should be considered in the genetic testing of myocarditis patients. Notably, the presence of HLA DQB1* MHC class II molecules has been linked to myocarditis and inflammatory cardiomyopathy.^107^ Subsequent studies have demonstrated the binding of MYH6 epitopes to HLA DQB1* molecules implicating a genetic predisposition in individuals expressing these HLA alleles.^17,21^ Although identifying an immune-genetic predisposition in myocarditis patients may refine diagnostic accuracy, inform risk stratification, and guide long-term management strategies, it is underutilized, in part due to cost, limited insurance coverage, and absence of clear consensus on which patients should be tested. Testing is most informative in cases with recurrent myocarditis, myocarditis with persistent cardiac dysfunction or sustained arrhythmias, family history of cardiomyopathy or sudden cardiac death, or histologic/electrocardiographic features suggestive of an inherited arrhythmogenic condition^3^ (*Figure 4*). Sensitivity is inherently limited—pathogenic variants are found in only a minority of unselected myocarditis patients, and interpretation of variants of uncertain significance requires specialized expertise. Moreover, access to genetic counsellors remains uneven across healthcare systems, impeding integration into routine care. Globally, genetic testing capacity is highly variable, further widening diagnostic disparities.^108^

## Treatment

Management of myocarditis is determined by the disease severity, clinical manifestations, and aetiology. Patients presenting with arrhythmias, heart blocks, or heart failure with reduced ejection fraction (HFrEF) should be treated according to established guidelines.^109–111^ Patients with complicated myocarditis often exhibit severely reduced LVEF and cardiogenic shock, necessitating inotropic support and vasopressors.^71^ For patients unresponsive to medical therapy, invasive interventions may be required including temporary mechanical circulatory support with percutaneous ventricular assist devices (e.g. Impella), veno-arterial extracorporeal membrane oxygenation, durable ventricular assist devices, or even heart transplantation.^71,93^ Notably, myocarditis patients undergoing heart transplantation are generally younger and more critically ill, often requiring mechanical support prior to the procedure; however, their long-term survival rates and post-transplant complications are comparable with those of patients transplanted for other aetiologies.^112,113^

Despite a lack of strong evidence supporting the use of corticosteroids in treating myocarditis, the first-line treatment for acute FM is often high-dose corticosteroids due to their potent immunosuppressive effects on T cell activation with reduction of antigen presentation, co-stimulation and pro-inflammatory cytokines^114^; however, other anti-inflammatory agents have been studied (*Table 1*). The trials summarized in *Table 1* collectively highlight the challenges of immunosuppressive therapy in myocarditis. Early studies, such as the Myocarditis Treatment Trial,^118^ failed to show benefit, likely due to delayed initiation of therapy (often >4 weeks from onset), inclusion of heterogeneous aetiologies, and limited mechanistic phenotyping. More recent trials (e.g. ARAMIS with anakinra^119^) have reinforced that broad inclusion criteria and small sample sizes can dilute potential treatment effects. Importantly, ongoing and upcoming studies such as MYTHS^120^ and COTAM^121^ are addressing these limitations by focusing on early intervention, standardized diagnostic criteria (often requiring CMR or biopsy confirmation), and stratification by disease severity (*Table 1*). These efforts reflect a shift towards precision immunosuppression—targeting pathways most relevant to the dominant immune mechanism (e.g. T cell-mediated inflammation in ICI myocarditis with abatacept, or type 2 immune responses in eosinophilic myocarditis with anti-IL-5 agents). The overall implication is that future immunosuppressive strategies should be guided by timely diagnosis, mechanistic profiling, and disease stage, ideally within adaptive trial designs that can test pathway-specific interventions in well-defined patient subsets. This approach is likely to improve the signal-to-noise ratio seen in past trials and enable the development of targeted therapies with higher efficacy and safety.

**Table 1:** Published and ongoing clinical trials in myocarditis

Name	ClinicalTrials.gov ID, sponsor (if applicable), and planned completion date (if applicable)	Type	Intervention	Outcomes	Results (if available)
Myocarditis Treatment Trial—A Clinical Trial of Immunosuppressive Therapy for Myocarditis^118^	N/A Completed	Randomized, investigator-initiated, controlled study	Azathioprine-prednisone group: Azathioprine 1 mg/kg ×2d for 24 weeks Prednisone 1.25 mg/kg ×1d in divided doses, then taper Cyclosporine-prednisone group: Cyclosporine: 5 mg/kg ×2d then tapered Prednisone: 1.25 mg/kg ×1d in divided doses	Primary: effect of treatment after adjustment for the LVEF and other characteristics at baseline	Does not support treatment of myocarditis with immunosuppressive drugs; high long-term mortality
ARAMIS (The Anakinra vs Placebo for the Treatment of Acute Myocarditis)^119^	NCT03018834 Assistance Publique—Hôpitaux de Paris Completed	Double blind, randomized controlled, multicentre, national, phase III trial	Placebo (*n* = 60) vs anakinra (*n* = 60) 100 mg ×14d	Primary: days alive free of any myocarditis complications Secondary: total quality adjusted life yearly (QALYs), LVEF by MRI, etc.	No difference in the number of days free of any myocarditis complications between the two arms (odds ratio for the composite outcome was 0.59)
COTAM (Corticoid Therapy in Acute Myocarditis)	NCT06522100 Assistance Publique—Hôpitaux de Paris 16 August 2028	Phase 3, multicentre, prospective, randomized, placebo controlled, double blinded	Placebo vs methylprednisolone IV ×3d followed by prednisone + conventional HF treatment	Primary: major cardiovascular events and/or persistence of LV dysfunction (EF < 50%) and/or GLS < −16% at 6 months	Recruiting
MYTHS (Myocarditis Therapy with Steroids)	NCT05150704 Niguarda Hospital December 2028	Phase 3, multicentre international, single blind, randomized	Placebo vs IV methylprednisolone 1 g ×3d	Primary: reduction in rate of all-cause death, heart transplant, LVAD implant, need for upgrading t-MCS, VT/VF, advanced AV block	Recruiting
MYTHS-MR (Myocarditis Therapy with Steroids in Patients with Mildly Reduced Ejection Fraction)	NCT05974462 University Hospital, Antwerp October 2028	Phase 3, multicentre, international, single blind, randomized	Placebo vs IV methylprednisolone 125 mg ×3d	Primary: LVEF ≥ 55% or an absolute increase in LVEF ≥ 10% on echocardiogram after 5 days	Recruiting
ARGO (Colchicine vs Placebo in Acute Myocarditis Patients)	NCT05855746 Assistance Publique—Hôpitaux de Paris July 2028	Phase 3, multicentre, prospective, randomized, double blind, placebo controlled	Placebo vs colchicine 0.5 mg BID ×6 months	Primary: extent of LGE evaluated on CMR and composite clinical primary outcome at 6 months	Recruiting
ACHLYS (Abatacept for the Treatment of Immune-Checkpoint Inhibitors Induced Myocarditis)	NCT05195645 Assistance Publique – Hôpitaux de Paris Completed	Double blind, randomized, dose-finding, multicentre, phase II trial	Arm A: Abatacept 10 mg/kg Arm B: Abatacept 20 mg/kg Arm C: Abatacept 25 mg/kg	Primary: proportion of patients with an adequate circulating monocytes CD86 receptor occupancy (CD86RO) saturation ≥80%	Unavailable
ATRIUM (Abatacept in ICI myocarditis)	NCT05335928 Massachusetts General Hospital April 2027	Phase 3, investigator-initiated, randomized, double-blind, placebo-controlled study	Placebo vs IV abatacept (10 mg/km) administered after randomization, then after 24 h, and finally14 days (optional 4th dose at 28 days)	Primary: measure any major adverse cardiac events (MACE)	Recruiting
HYPIC (The Effects of Hydroxychloroquine in Patients with Inflammatory Cardiomyopathy)^122^	NCT05961202 Tongji Hospital November 2025	Phase 3, multicentre, randomized	Hydroxychloroquine 200 daily + prednisolone 20 mg daily for 12 m vs prednisolone 20 mg daily for 12 months	Primary: composite cardiovascular outcome at 3 years (time to death or heart transplant, hospitalization for heart failure or recurrence of myocarditis, permanent pacemaker, or ICD implant)	Recruiting
TRINITY (Immunosuppressive Treatment in Chronic Virus-Negative INflammatory Cardiomyopathy)	NCT05570409 LMU Klinikum March 2028	Phase 3, multicentre, randomized, double-blind, placebo-controlled	Mycophenolate mofetil 1 g BID ×6 months + prednisolone 1 mg/kg initially then taper over 6 months vs mycophenolate and prednisolone placebo	Primary: Absolute increase in LVEF and proportion of patients with an absolute increase in LVEF ≥ 10% at 12 months	Recruiting

Description of the completed and ongoing trials investigating therapies for myocarditis. This table provides several important insights into myocarditis management. (i) There is a paucity of clinical trial evidence informing clinical management. (ii) Prior randomized controlled studies using immunosuppression for myocarditis have failed to show significant improvement in their primary endpoints. (iii) There are multiple ongoing efforts to investigate therapeutic strategies in this space and may provide better clarity on management in the near future.

Beyond medical treatments, device-based interventions can be beneficial for certain patients with myocarditis. In cases of FM, severe conduction abnormalities such as second- and third-degree atrioventricular (AV) blocks are common, making temporary or permanent pacing advisable. For patients experiencing ventricular arrhythmias, cardiac sarcoidosis, or giant cell myocarditis, an implantable cardioverter-defibrillator (ICD) should be considered, given that acute myocarditis accounts for approximately 6%–10% of sudden cardiac deaths in young adults.^123–125^

Patients with myocarditis are advised to refrain from intense physical activity for at least 1 month (extended depending on the initial presentation) and should undergo evaluations to monitor for remission of myocardial inflammation on CMR.^3^ A gradual return to physical activities is appropriate once LVEF and troponin levels have normalized, and no clinically significant arrhythmias are detected during monitoring.^126^

In addition to the general guidelines and recommendations for the treatment of myocarditis, which apply to the full range of disease manifestation (*Figure 3*), specific disease mechanisms or particularly fulminant disease courses require the implementation of special treatment procedures.

### Immune checkpoint inhibitor myocarditis

In patients with ICI myocarditis, the inciting immunotherapy should be discontinued promptly, and high-dose intravenous corticosteroids should be initiated.^1^ Earlier initiation (within the first 24 h) and high-dose corticosteroids are associated with an improved outcome.^115^ Alternatively, global T cell depletion with alemtuzumab (anti-CD52 antibody) or antithymocyte globulin (anti-CD3 antibody) have been used to treat myocarditis.^116,117,127^ In cases refractory to steroids, other immunomodulators could be used to reverse the effects of ICI and to inhibit T cell activation by blocking co-stimulatory molecules (*Figure 2C*). In a small cohort study of 30 patients with ICI myocarditis, the combination of abatacept (a CTLA-4 antagonist) and ruxolitinib (a Janus kinase inhibitor) led to a dramatic decrease in mortality rate, from 60% to only 3% of patients.^128^ Ongoing clinical trials are currently evaluating the efficacy of abatacept in ICI myocarditis (NCT05335928).

### Giant cell myocarditis

In patients with giant cell myocarditis, which often presents with rapid haemodynamic deterioration and malignant arrhythmias, immunosuppression with high-dose intravenous methylprednisolone should be initiated promptly.^1,4,129^ Maintenance treatment with corticosteroids, antithymocyte globulin, and cyclosporine may halt disease progression in two-thirds of patients with giant cell myocarditis, according to case series.^113,130,131^ Clearly, future clinical trials should address more specific and targeted immunotherapeutical approaches to attenuate immune cell activation in general and to reduce T cell activity in the myocardial microenvironment.

### Eosinophilic myocarditis

Patients with eosinophilic myocarditis are treated with systemic glucocorticoids with or without additional immunosuppressive agents such as azathioprine or cyclosporine.^3^ A meta-analysis showed lower in-hospital mortality in patients treated with corticosteroids.^129^ A case report also indicated successful treatment of eosinophilic myocarditis with interleukin-5 inhibitor, mepolizumab.^132^ Since eosinophilic myocarditis belongs to the extended group of type 2 immunity-related diseases,^133^ immunotherapeutical interventions targeting specific T helper 2 cell-derived factors will most likely become therapeutic options for eosinophilic myocarditis.

### Follow-up and surveillance

Following an acute episode of myocarditis, three potential clinical trajectories exist: complete remission, progression to chronic myocarditis, or recurrence (*Figure 3*). Regular surveillance, including clinical assessment, laboratory tests, exercise testing, as well as repeat echocardiography and CMR within 6 months of hospitalization, is thus crucial to guide management and assess long-term outcomes. For patients with persistent structural abnormalities, repeated imaging after 6 months is recommended to monitor disease progression and guide therapeutic decisions. In those with complete recovery of LVEF, the safety of GDMT tapering remains uncertain. Current recommendations advise continuation of HF therapy for at least 6 months upon complete recovery of cardiac function. Prospective registries and clinical trials are essential to inform long-term management strategies.

## Future directions and research opportunities

Despite advances in our understanding of myocarditis, significant gaps remain across mechanistic, diagnostic, and therapeutic domains. Future research should prioritize the following directions.

### Enhancing reverse translational research

Much of our mechanistic understanding derives from animal models, which incompletely recapitulate the human immune landscape. Leveraging transcriptomic profiling—both in peripheral blood and myocardial tissue—can provide high-resolution insight into immune activation patterns, cellular interactions, and gene expression signatures that define human myocarditis subtypes.^20^ Integration with proteomic and spatial transcriptomic approaches may help resolve tissue-level heterogeneity and immune cell localization, especially when linked to clinical phenotypes.

### Expanding the spectrum: emerging forms of myocarditis

Novel aetiologies of myocarditis are increasingly recognized, including cases associated with mRNA-based vaccines, ICIs, and, more recently, gene therapies.^70,134^ These distinct forms raise important questions about immune tolerance, antigen mimicry, and T cell cross-reactivity. Dedicated study of these entities will be critical, not only to improve diagnosis and treatment but also to illuminate shared and divergent pathways across myocarditis types.

### Genetic underpinnings and therapeutic implications

Recent work has implicated genetic susceptibility—particularly in immune regulation and structural myocardial genes—in predisposing individuals to myocarditis.^99,102^ Future research should explore how host genetics modulate disease onset, severity, and recovery and assess the potential for gene-modifying or gene-silencing therapies in selected cases. Moreover, given the growing evidence linking specific HLA alleles to myocarditis,^17,21,107^ further investigation is warranted to clarify their role in susceptibility to myocardial inflammation and to evaluate their potential application in risk stratification and therapy.

### Improving diagnosis: biomarkers and imaging

Diagnosis remains a major challenge given the non-specific clinical presentation and reliance on invasive biopsy (*Table 2*). Circulating biomarkers that reflect immune activation or myocardial injury with greater specificity are urgently needed. Molecular imaging techniques, including immuno-PET, hold promise for non-invasively visualizing myocardial inflammation and identifying specific immune cell subsets *in vivo*. Furthermore, future studies are needed to link complex immunological biomarker profiles with clinical phenogroups to support clinical decision-making. Strategies that can be already implemented in clinical practice involve performing of serological immunophenotyping prior to initiating immune-targeted therapies. By measuring the specific immunological molecules targeted by the therapeutic agent, this approach may support clinical decision-making by aiding in the identification of the most appropriate treatment strategy.

**Table 2:** Strengths and weaknesses of diagnostic modalities

	Endomyocardial biopsy	Cardiac MRI	Genetic testing
Strengths	Allows for determination of histologic subtype of myocarditis including viral or immune mediated with subtyping for lymphocytic, eosinophilic, giant cell, and sarcoidosis For high-risk patients, can be performed in concert to other procedures in the catheterization lab including coronary angiogram, RHC, or MCS placement Early EMB in fulminant myocarditis has been shown to correlate with lower rates of death, transplant, and LVAD at 1 year	Provides better myocardial definition than echocardiography Allows for visualization of myocardial inflammation (T1 weighting), oedema (T2 weighting), and fibrosis (LGE) Can assist in ruling out other forms of cardiomyopathy including coronary ischaemia Distribution of LGE can inform prognosis (mid-wall and anteroseptal LGE are associated with worse prognosis)	Identification of high-risk variants can inform prognosis and, in some cases, may change management strategy (e.g. ICD placement in ARVC) Provides important information for family planning and screening of family members
Limitations	Invasive and associated with a small but serious risk for complication Significant interobserver variability Quantitative parameters for cell counts are currently being debated Varying sensitivity based on myocarditis type (highly sensitive in GCM but limited in sarcoid) Lack of widespread molecular viral testing and immunohistochemical analyses	Diagnostic accuracy varies based on clinical presentation and timing Does not perform well in ICI myocarditis or in chronic myocardial inflammation Limited in patients with metallic implants Relies on quality of healthcare system’s MRI capacities	Expensive and often poorly covered by insurance Lack of consensus as to ideal candidates for testing Requires access to specialized healthcare infrastructure including genetic counsellors Often limited to a small set of established cardiomyopathy mutations (limited assessment of possible immunologic drivers)
2025 ESC guidelines	Recommended in high-risk patients or in intermediate risk when knowing the histologic type could aid in management Class I LOE C for EMB in high-risk myocarditis including haemodynamically instability and in intermediate risk patients not responding to conventional therapy to detect histologic subtype that could influence management	Emphasized growing importance in the newest guidelines (suggesting that it could eliminate the need for EMB in many cases) Recommended in low/intermediate risk patients Class I LOE B for patients with suspected myocarditis Class I LOE C for follow-up MRI within 6 months of diagnosis to assess resolution, risk stratification, and return to exercise	Class I LOE C recommendation to obtain a family history including pedigree in patients with recurrent myocarditis Class IIa LOE B for genetic testing in patients with definite myocarditis with family history, ventricular arrhythmia, significant LGE, recurrent disease, or persistent troponin elevation

The strengths and weaknesses of diagnostic modalities in myocarditis. This table provides practical considerations for the main modalities currently used to investigate patients with myocarditis. For example, although the ESC guidelines have emphasized the use of cardiac MRI in the diagnosis of myocarditis, there remain practical barriers relevant to MRI capabilities of each healthcare system that may limit a practitioner from using it to its full potential. Similarly, although endomyocardial biopsy can provide important information about the histologic subtype of myocarditis, there remains significant variability in the availability of viral testing and immunophenotyping. Finally, genetic testing has been recommended in a subset of patients with myocarditis including those with recurrent myocarditis but is often limited to a subset of cardiomyopathy related genes. The relevant ESC guidelines are highlighted for each modality.

### Targeted therapeutics: a T cell–centric approach

As T cells emerge as central players in the pathogenesis of many forms of myocarditis, there is growing interest in therapies that modulate T cell activation, trafficking, or specificity. Pre-clinical and early clinical studies should evaluate agents that selectively target autoreactive or cytotoxic T cells while preserving protective immunity. This is already the case with ICI myocarditis, where abatacept (CTLA-4Ig) has been successfully tested in pre-clinical models, as well as a case series of patients.^116,128,135^ As peripheral tolerance mechanisms are critical in controlling T cell-driven autoimmunity, enhancing the protective effect of T reg cells, for example, through complexed IL-2, may offer a potential treatment approach for both acute and chronic myocarditis.^136^ Adoptive transfer strategies of T reg cells, checkpoint modulation, and antigen-specific tolerance induction represent potential avenues for exploration. The heterogeneity of clinical manifestations of myocarditis poses significant challenges in designing clinical trials that identify patients most likely to benefit from treatment.

### Heterogeneity in clinical course: subsets of disease

Not all myocarditis is acute and self-limited. A subset of patients experiences recurrent or chronic disease, which may reflect underlying autoimmune predisposition or failure to resolve inflammation. Defining clinical and molecular phenotypes of myocarditis—including predictors of recurrence or progression to dilated cardiomyopathy—will be crucial to guide long-term management and follow-up. Advances in patient immunoprofiling are expected to facilitate the development of clinical trials with more targeted therapeutic approaches.

### Infrastructure for progress: multi-institutional collaboration and biobanking

Progress in myocarditis research has been hampered by small, heterogeneous cohorts. Establishing large-scale, prospective biobanks with standardized phenotyping, biospecimen collection, and imaging will enable robust, reproducible discoveries. Multi-institutional consortia are essential to provide the power and diversity needed to uncover meaningful subtypes, validate biomarkers, and test novel therapies in well-characterized populations.

## Key advances and conclusions

Here we review the latest clinical appreciation of myocarditis through the pathophysiological lens of a breakdown in immune tolerance, focusing on the critical role T lymphocytes play in myocarditis. We provide a novel perspective by integrating existing myocarditis models into an overarching immunological concept centred on T cell tolerance and regulation. This concept focuses on immunological mechanisms and will inform the immunophenotyping of patients as a future diagnostic tool. It will also guide the design of clinical trials for new immune-modulating treatment strategies. Emerging forms of myocarditis, including myocarditis associated with immune-based cancer therapies, vaccines, and gene therapies, add further urgency to developing novel diagnostic and therapeutic strategies. New technological advances in immunology allow for better interrogation of underlying mechanisms of cardiac inflammation in general, but especially in the case of myocarditis.

## Declarations

### Data Availability

No data were generated or analysed for or in support of this paper.
